# Screening of Bovine Tissue-Specific Expressed Genes and Identification of Genetic Variation Within an Adipose Tissue-Specific lncRNA Gene

**DOI:** 10.3389/fvets.2022.887520

**Published:** 2022-05-11

**Authors:** Sihuan Zhang, Han Xu, Enhui Jiang, Zhanerke Akhatayeva, Fugui Jiang, Enliang Song, Chuanying Pan, Hong Chen, Xianyong Lan

**Affiliations:** ^1^College of Animal Science and Technology, Northwest A&F University, Xianyang, China; ^2^School of Medicine, Sun Yat-sen University, Guangzhou, China; ^3^Institute of Animal Science and Veterinary, Shandong Academy of Agriculture Science, Jinan, China

**Keywords:** cattle, transcriptome, adipogenesis, adipose tissue-specific genes, insertion/deletion

## Abstract

Global classification of bovine genes is important for studies of biology and tissue-specific gene editing. Herein, we classified the tissue-specific expressed genes and uncovered an important variation in the promoter region of an adipose tissue-specific lncRNA gene. Statistical analysis demonstrated that the number of genes specifically expressed in the brain was the highest, while it was lowest in the adipose tissues. A total of 1,575 genes were found to be significantly higher expressed in adipose tissues. Bioinformatic analysis and qRT-PCR were used to uncover the expression profiles of the 23 adipose tissue-specific and highly expressed genes in 8 tissues. The results showed that most of the 23 genes have higher expression level in adipose tissue. Besides, we detected a 12 bp insertion/deletion (indel) variation (rs720343880) in the promoter region of an adipose tissue-specific lncRNA gene (*LOC100847835*). The different genotypes of this variation were associated with carcass traits of cattle. Therefore, the outcomes of the present study can be used as a starting point to explore the development of cattle organs and tissues, as well as to improve the quality of cattle products.

## Introduction

Cattle (*Bos taurus*) is one of the most important livestock species in the world, which was domesticated about 10,500 years ago, at least since the early Neolithic period (1). Nowadays, cattle are commonly raised for beef, milk, and hides. Compared to other types of livestock, cattle produce larger quantities of healthy and palatable meat and milk, which play a critical role in providing the population with a complete balanced diet, as well as occupying an important position in the global livestock economy. However, researches on gene expression profiles in cattle have lagged behind compared to humans and mice, Many databases, such as NCBI and RNA-Seq Atlas can be used to view the human and mouse gene expression profiles (2), but there are few studies on bovine gene expression profiles in multiple tissues (3–5). Since global and tissue-specific expression of genes are the main causes leading to differences in biological phenotype, organism and tissue functions, tissue-specific expression analysis of bovine genes is of great significance for studying the biological functions of bovine genes.

In cattle, there are four main categories of adipose tissue: visceral adipose, subcutaneous adipose, intramuscular adipose, and intermuscular adipose tissues. Different adipose tissues have different functions. The deposition of intramuscular adipose and intermuscular adipose is closely related to the sensory qualities of beef, such as palatability, tenderness, juiciness, and flavor (6). The deposition of subcutaneous adipose and visceral adipose is directly related to feed conversion rate and carcass traits, and excessive deposition reduces productivity and economic efficiency. Deposition of adipose in different parts of the body is a consequence of different gene expression (7). Therefore, the study of gene expression patterns in different adipose tissue is important to explore the mechanism of adipose-specific deposition and improve the production efficiency and beef quality.

Adipogenesis is a complex and delicate process, which is controlled by numerous factors, such as hormones and transcription factors (8). Accumulating evidence indicates that hormones, including glucocorticoids, insulin, thyroid hormone, leptin, and TNF-α play crucial roles in adipogenesis (9, 10). These hormones jointly regulate this process via autocrine and paracrine action (11). Furthermore, transcription factors control adipogenesis through the sequential activation of numerous genes associated with adipogenesis, such as the well-known peroxisome proliferator activated receptor gamma (PPARγ), CCAAT/enhancer-binding protein (C/EBPs), and sterol-regulatory element binding proteins (SREBPs) (12, 13). Activated transcription factors and their downstream genes form a regulatory network that cooperatively regulates the development of adipose tissue (12, 13). This well-organized and multi-step process involves many genes. Therefore, the identification of adipogenesis related genes and the construction of an interaction network are essential for a comprehensive understanding of bovine adipogenesis.

Due to the rapid development of high-throughput sequencing technology and the continuous improvement of publicly available animal databases (14), it has become possible to obtain large amounts of RNA sequencing data and identify bovine tissue-specific genes. In this study, for the first time, we systematically identified bovine tissue-specific genes, especially adipose tissue-specific genes (ATSGs). Besides, the genes that significantly higher expressed in bovine adipose tissue (ATHEGs) were also identified. Furthermore, this study identified an important variation in an adipose tissue-specific lncRNA gene, which was associated with carcass traits and can be used in cattle breeding. The results of this study will contribute to a systematic understanding of the bovine genes expression characteristics and provide a basis for improving the quality and yield of beef.

## Materials and Methods

This study was carried out in accordance with the recommendations of the Institutional Animal Care and Use Committee (IACUC) of Northwest A&F University (NWAFU-314020038), Yangling, Shaanxi, China.

### Data Collection

This study collected 65 RNA sequencing data sets from 14 tissues (visceral adipose tissue, subcutaneous adipose tissue, intramuscular adipose tissue, muscle, heart, liver, spleen, lung, kidney, skin, brain, cerebellum, pituitary, and small intestine) of cattle (*Bos Taurus*). All these data were downloaded from NCBI GEO database. In order to identify important variations in adipose tissue-specific genes, this study collected and isolated DNA samples from 516 Shandong black cattle (with corresponding data of carcass traits, including the weight of cervical bone, bull collar, tendon, round small intestine, topside, hollow bone, rump steak, chuck tender, rib eye, tender loin, beef brisket, flank, chuck roll, and striploin), 59 Luxi cattle, 60 Bohai black cattle, and 37 Mengshan cattle in Shandong province, China.

### RNA-Seq Data Processing

The quality of raw sequencing data was assessed using FastQC v0.11.8 (http://www.bioinformatics.babraham.ac.uk/projects/fastqc/) (15). In order to obtain clean reads, the sequencing data were filtered. The connectors at both ends of reads were removed, and the sequences containing low-quality bases were cut off. The clean reads were compared to the bovine reference genome (GCF_002263795.1_ars-UCD1.2_genomic.fna) using HISAT2 version 2.1.0 (16). The expression level (fragments per kilobase of transcript per million, FPKM) of all genes was calculated based on the bovine genome file (GCF_002263795.1_ars-UCD1.2_genomic.gFF) (15). A principal components analysis (PCA) was then performed, and some outliers were removed. Finally, 52 data sets were reserved for subsequent analysis, and each tissue containing 2–11 SRA data sets (Supplementary Table 1).

### Identification of Tissue-Specific Genes

Since many genes were not expressed or had very low expression in the sequencing data collected in this study, genes with FPKM <1 in all tissues were removed, and a total of 20,446 genes were left after filtering. Then the tissue-specific index τ of each gene was calculated (17, 18). The τ value varies from 0 to 1. A τ value close to 1 indicates high tissue-specificity of gene expression, while τ value close to 0 indicates that the gene is widely expressed in all tissues (17, 18). For tissues with multiple biological replicates, the average value of FPKM values was used. Next, the top 20% genes were selected as candidate tissue-specific genes according to τ values. If the FPKM value of a candidate gene in a tissue is more than 1, and the expression level in that tissue is among the top three of all detected tissues, the candidate gene will be defined as a tissue-specific gene (19).

### Identification of Genes Significantly Higher Expressed in Adipose Tissues

HTSeq Version 0.11.2 software was used to calculate the number of reads mapped to each gene according to the bam files generated after comparison (20). Subsequently, differential expression of genes was analyzed by DEseq2 package in *R* (*P* adjust < 0.05).

### Functional Annotation of Candidate Genes

The KOBAS (http://kobas.cbi.pku.edu.cn/kobas3) was utilized for Gene Ontology (GO) clustering analysis and Kyoto Encyclopedia of Genes and Genomes (KEGG) enrichment analysis of candidate genes.

### Total RNA Isolation and cDNA Synthesis

The bovine samples used for gene expression profile analysis were obtained from Kingbull Livestock Co., Ltd., (Yangling, Shaanxi, China). Tissues (spleen, lung, kidney, muscle, visceral fat, brain, testis, ovary) from four calves (two male and two female) were collected. RNA was isolated from tissue samples using Trizol reagent (Takara, Dalian, China). Consequently, RNA was used as template to prepare cDNA using Prime Script™ RT Reagent kit (Takara, Dalian, China). The prepared cDNA was preserved at −20°C.

### Gene Expression Profile Analysis

The PCR and agarose gel electrophoresis were used to determine in which tissues the gene is expressed. Then, genes expressed in more than four tissues were selected for quantitative reverse transcription-polymerase chain reaction (qRT-PCR) assay to analyze their expression levels in different tissues. The primers were designed using NCBI Primer-BLAST (Table 1). The 25 μL reaction system contained 12.5 μL 2 × ChamQ SYBR qPCR Master Mix (Vazyme biotech co., ltd. Nanjing, China), 10 ng cDNA, 5 pM of each primer. The reaction procedure is as follows: pre-denaturation at 95°C for 45 s; 40 cycles of denaturation at 95°C for 15 s, annealing and elongation at 60°C for 40 s. The 2^−Δ*ΔCt*^ method was applied to compute the relative gene expression levels in tissues (21). The *glyceraldehyde-3-phosphate dehydrogenase* (*GAPDH*) gene was employed as an internal control.

**Table 1:** Primers used in this study.

**Gene names**	**Forward primer sequence**	**Reverse primer sequence**	**Product sizes**
ADAMTS16	CGATGCTACGGAAGATGCCT	CGCACGGAAATGTAGGAGGT	183 bp
ADIG	CACTGGTGAACGAGCTGACAT	TGAGTAAGAAGCGTAGCCAGA	102 bp
COMP	GCAGAAAGGACAACTGCGTG	CGTATTTCGCTGGTCTGGGT	158 bp
GSC	CAGGAGACCAAGTACCCAGA	CTTGTTCCACTTCTCAGCGT	165 bp
GIPR	TTGGCATCCTCGTGTCAAAG	GGAAGCCCTGGAAAGAACTGA	207 bp
EN1	GGCCATACTGCTAATGGGCT	ATTCCGCCTTGAGTCTCTGC	221 bp
HCAR1	TATTCCTTGTCGGGTGGTGC	GACACAGACAATGCCAACCG	160 bp
KERA	TCCCCCATATATAGCACAGCC	AGCTGTGAGAGAGACAAAAAGGA	103 bp
LHCGR	CCATCTCAAGCTTTCAGAGGAC	TTGAGGAGGTTGTCAAAGGCA	104 bp
LEP	TGCCCATCGACAGGCCTCACT	CTGGATCTCCCCCGCTCCCA	254 bp
LOC101907335	GGGGAATGAGGAGGAAGTGC	TATCTGAGGCTTCATCTCCTAGT	238 bp
LOC107131843	ATCAGAGTTAGCCGGGGAGT	CACGTAGTTGAGGCCTTGGT	102 bp
APOL6	TCACTGGAACCTGCTCAACC	GCCTTGACTGGGGCACTATT	255 bp
MDFIC2	TCAATGTTTGCTGCCCCTCT	GCCTGAACACGGTTCCTTTG	195 bp
S100A5	TTCTCTGGCTCTCTCCTTCCTTG	ATCTCCTGGTCGCTGTTCTT	247 bp
TRARG1	TCTCGAAGTAGCGTGCAACA	GCACCGCTCCCTAGTTAGTT	167 bp
LOC100847835	TCCCGTCTCACCCCTACATC	CTTGACCCACAGGGACTGAC	230 bp
LOC104969981	ATCTGGATTCGACTGCACCC	GACCCTTGTTCTTCGTTTGCT	99 bp
LOC104970976	AACAAGGGCTGAATCTTGGC	CCCCGGAGCATCTCATTTCA	201 bp
LOC112446369	TGCTCCGTCCTGATTGGATG	GCAGAGGGTGTTAGGATGGTC	201 bp
LOC112448034	CCCATTTGAAGGAGGGGCAA	ATGGGGGTCAACTTGGTGTG	217 bp
LOC112448366	TGGCCCCACTGCTCTTTACT	TCGCCGTAAGTTTGGTTGCT	256 bp
LOC112449245	GCAGGGTGTGTGATGCTACT	TAGCCAATCCTCTGGGCTCT	201 bp
rs720343880−1	GCCTCCTGATGAACTGGGTG	CCCGGAAGCCATACAGAAGA	216 bp
rs720343880−2	ATGGAGTCCTTGCAGGTTGG	GTCTGCTTCCTGCCTCTACC	216 bp

### Screening and Identification of Crucial Genetic Variation

The candidate important genetic variations of adipose tissue-specific genes were selected from the Ensembl database. Two pairs of primers were designed using NCBI Primer-BLAST to detect the authenticity and identify frequencies of the candidate variation (rs720343880) in different cattle breeds (Table 1). The touchdown-PCR method and 3.5% agarose gel electrophoresis were utilized to identify the genotypes of individuals. Sanger sequencing was used to confirm the accuracy of genotyping. Genetic parameters were computed on the MSRcall website (http://www.msrcall.com) according to the methods described as Nei (22), including observed heterozygosity (*Ho*), expected heterozygosity (*Ne*), polymorphism information content (*PIC*), and Hardy-Weinberg equilibrium (*HWE*) (22). The association analysis was performed based on the model similar to that in a previously published study (23). Significance between different genotypes and carcass traits was determined using an independent-sample *t*-test (the number of individuals in a group of less than three was not calculated).

## Results

### Preliminary Analysis of Data Sets

**Figure 1:**
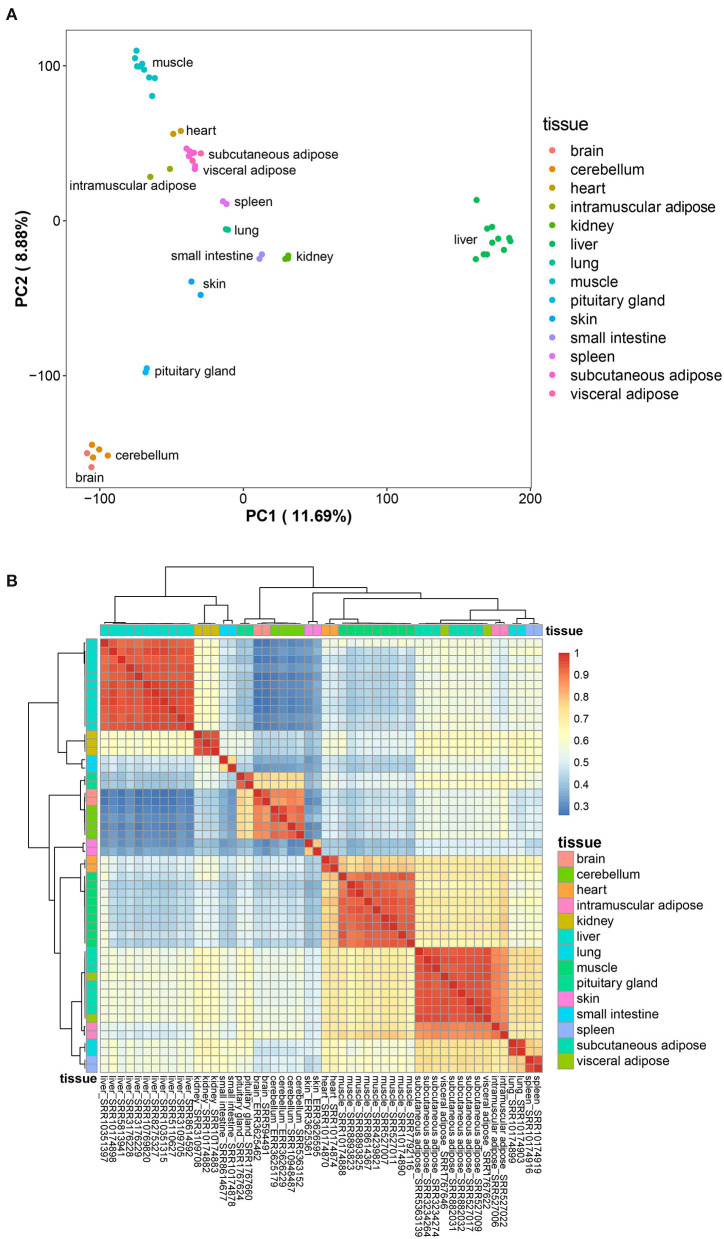
Preliminary analysis of data sets. **(A)**, Principal component analysis result of the data sets; **(B)**, the expression profile of the tissue-specific expressed genes.

Principal component analysis was carried out on the collected transcriptome data to eliminate outlier data sets. The remaining 52 data sets were analyzed, and the results showed that the rest of the data sets replicated well (Figure 1A). After removing genes with low expression in all tissues (FPKM < 1), a total of 4,090 genes with tissue-specific expression were detected. At the same time, cluster analysis based on the level of tissue-specific gene expression showed that different data sets from the same tissue aggregated well, and the three adipose tissues (subcutaneous adipose, visceral adipose, and intramuscular adipose tissues) clustered together (Figure 1B). The above results indicated that the data sets could represent the gene expression levels in different tissues, which can be used for subsequent analysis.

### Analysis of the Bovine Tissue-Specific Gene Characteristics

**Figure 2:**
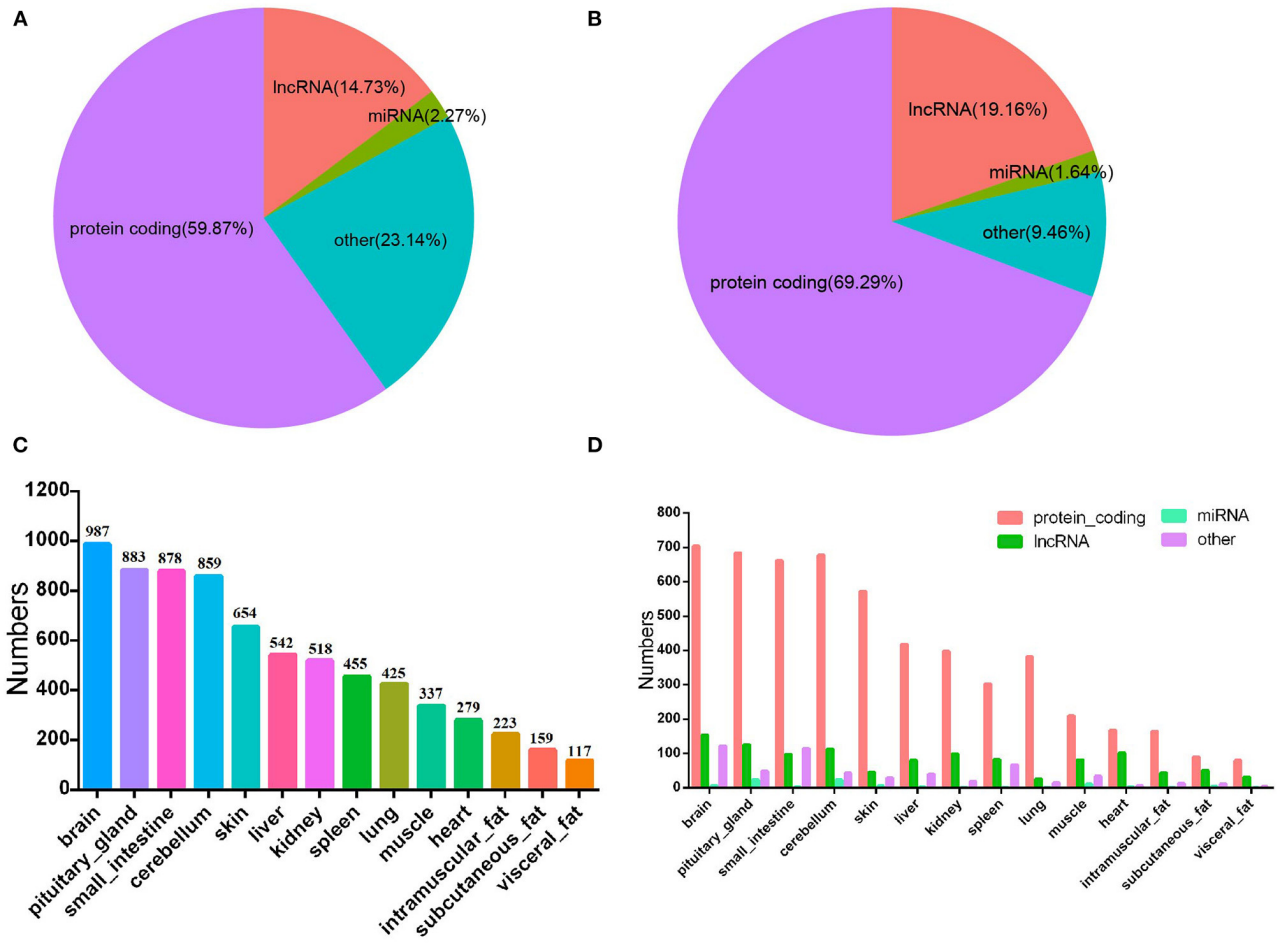
Characteristics of bovine tissue-specific genes. **(A)**, The distribution of different kinds of RNA gene of bovine genome; **(B)**, bovine tissue-specific genes; **(C)**, the numbers of bovine tissue-specific genes; **(D)**, the number and rate of each kind of the bovine tissue-specific genes.

To date, a total of 35,158 genes have been annotated in the bovine genome. Analysis of bovine transcriptome annotation data showed that 58.87% of the genes are protein-coding genes, 14.73% are lncRNA genes, 2.27% are miRNA genes, and other RNA genes (including tRNA genes, pseudogenes, piRNA genes, etc.) account for 23.14% (Figure 2A). Among the 4,090 tissue-specific genes, 69.29, 19.61, 1.64, and 9.46% were annotated as protein-coding genes, lncRNA genes, miRNA genes, and other RNA genes, respectively (Figure 2B). These results indicated that lncRNA have higher tissue expression specificity (3).

### Number of Bovine Tissue-Specific Genes

Results of statistical analysis showed the number of genes specifically expressed in tissues, including brain (*n* = 987), pituitary gland (*n* = 883), small intestine (*n* = 878), cerebellum (*n* = 859), skin (*n* = 654), liver (*n* = 542), kidney (*n* = 518), spleen (*n* = 455), lung (*n* = 425), muscle (*n* = 337), heart (*n* = 279), intramuscular adipose tissue (*n* = 223), subcutaneous adipose tissue (*n* = 159), and visceral adipose tissue (*n* = 117) (Figure 2C; Supplementary Table 2). In all tissues, protein-coding genes were the main gene type (Figure 2D). Tissue-specific expressed genes may be related to tissue multifunctionality, tissue-specific function and cell type. These tissue-specific expressed genes are crucial for revealing the characteristics of different tissues and the functions of different genes (24, 25).

**Figure 3:**
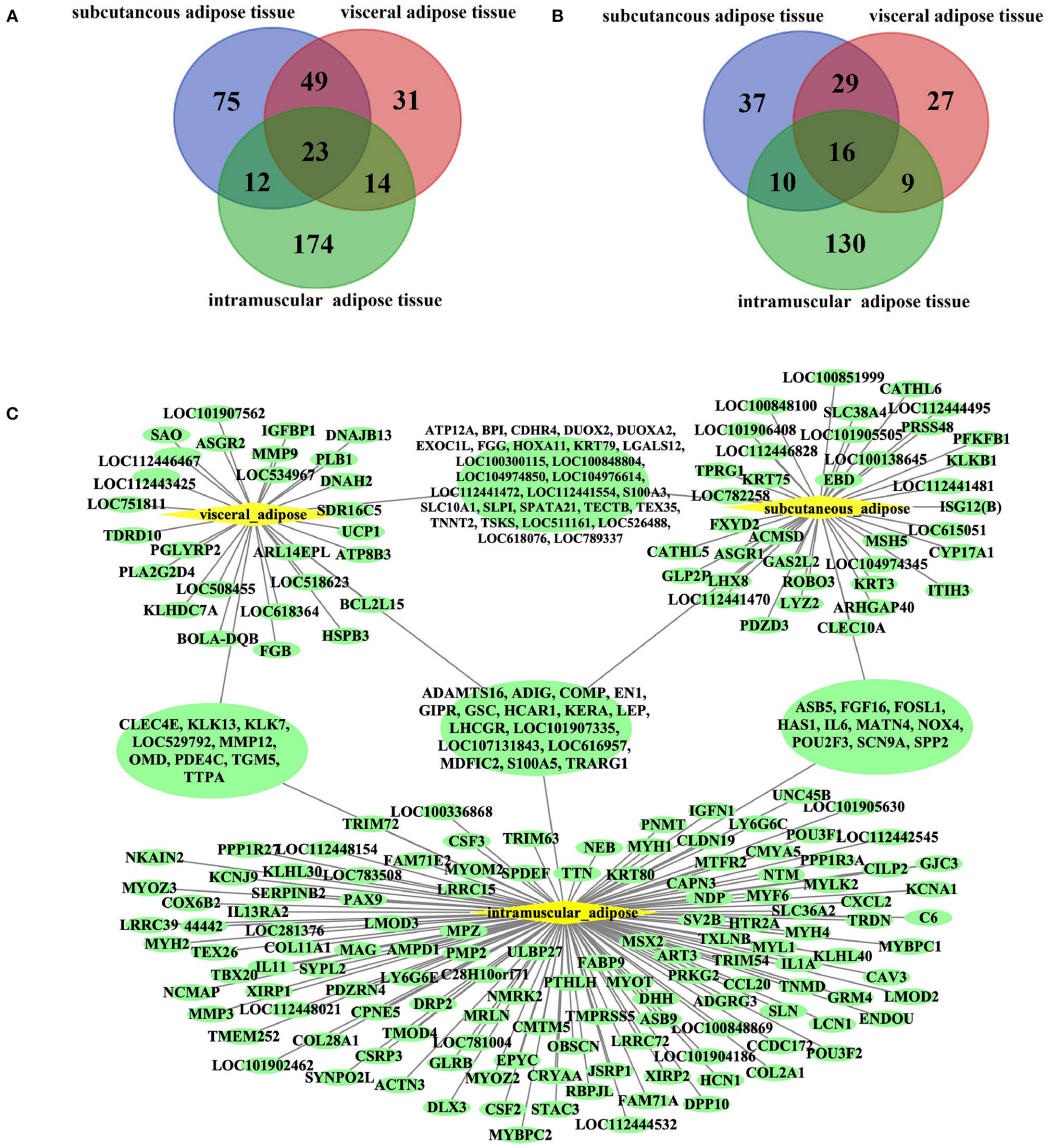
Bovine adipose tissue-specific expressed genes. **(A,B)**, The venn of adipose tissue-specific genes and protein-coding genes of three kinds of adipose tissues; **(C)**, tissues specific protein-coding genes of three kinds of adipose tissues.

Due to the essential role of fat in cattle development and beef quality, we focused on genes specifically expressed in adipose tissue. Among the ATSGs, we found 23 genes specifically expressed in all three adipose tissues, including 16 protein-coding genes and seven lncRNA genes (Figure 3). The 16 protein-coding genes are as follows: *ADAMTS16* (*ADAM metallopeptidase with thrombospondin Type 1 Motif 16*), *ADIG* (*Adipogenin*), *COMP* (*cartilage oligomeric matrix protein*), *EN1* (*Engrailed 1*), *GIPR* (*Gastric inhibitory polypeptide receptor, or glucose-dependent insulinotropic peptide*), *GSC* (*Goosecoid homeobox), HCAR1 (Hydroxycarboxylic acid receptor 1*), *KERA* (*Corneal protein*), *LEP* (*Leptin*), *LHCGR* (*luteinizing hormone/choriogonadotropin receptor*), *LOC101907335* (*antigen WC1.1-like*), *LOC107131843* (*insulin receptor substrate 1-like*), *LOC616957* (*apolipoprotein L6, APOL6*), *MDFIC2* (*MyoD Family Inhibitor Domain containing 2*), *S100A5* (*S100 Calcium binding protein A5*), and *TRARG1* (also known as *SLC2A4* (*Solute carrier family 2 member 4*) and *GLUT4* (*Glucose transporter type 4*). The 7 lncRNA genes are *LOC100847835, LOC104969981, LOC104970976, LOC112446369, LOC112448034, LOC112448366*, and *LOC112449245*. There were 49 genes belonged to subcutaneous adipose tissue-specific genes (SATSGs) and visceral adipose tissue-specific genes (VATSGs), including 29 protein-coding genes, 18 lncRNA genes, one pseudogenes, and one miRNA gene (*MIRN214*) (Figure 3). Moreover, SATSGs and intramuscular adipose tissue-specific genes (IATSGs) included 12 genes: 10 protein-coding genes and two lncRNA genes (Figure 3). In addition, there were 14 genes belonged to IATSGs and VATSGs, including nine protein-coding genes, four lncRNA genes, and one pseudogene (Figure 3). Further statistical analysis showed that a total of 378 genes and 257 protein-coding genes were specifically expressed in adipose tissue (Figure 3).

### The Expression Characteristics of Bovine Adipose Tissue-Specific Genes

**Figure 4:**
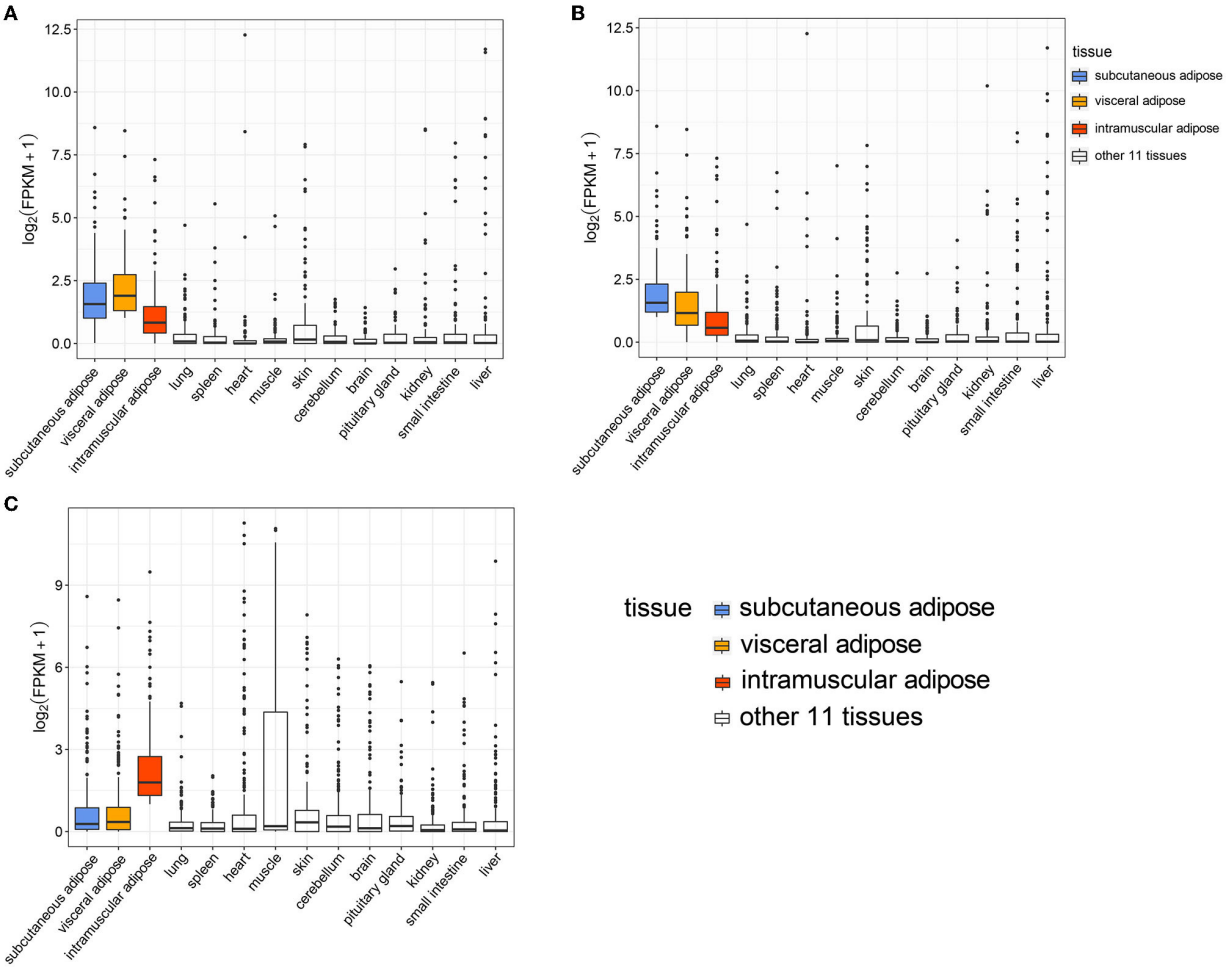
The expression level of visceral adipose **(A)**, subcutaneous adipose **(B)**, and intramuscular adipose **(C)** tissue-specific genes in 14 tissues.

Analysis of the expression levels of genes specifically expressed in visceral adipose tissue (VAT) in all tissues showed that the expression level of these genes was highest in VAT, followed by two other types of adipose tissue, subcutaneous adipose tissue (SAT) and intramuscular adipose tissue (IAT) (Figure 4A). Analysis of the expression levels of genes specifically expressed in SAT in all tissues exhibited that the expression level of these genes was greatest in SAT, followed by the other two types of adipose tissues (Figure 4B). The expression level of genes specifically expressed in IAT was the highest in IAT, but was lower in the other two adipose tissues, indicating that the gene expression patterns of VAT and SAT were more similar (Figure 4C). Besides, many IATSGs were highly expressed in muscle, suggesting that gene expression patterns between IAT and muscle may be more similar (Figure 4C).

**Figure 5:**
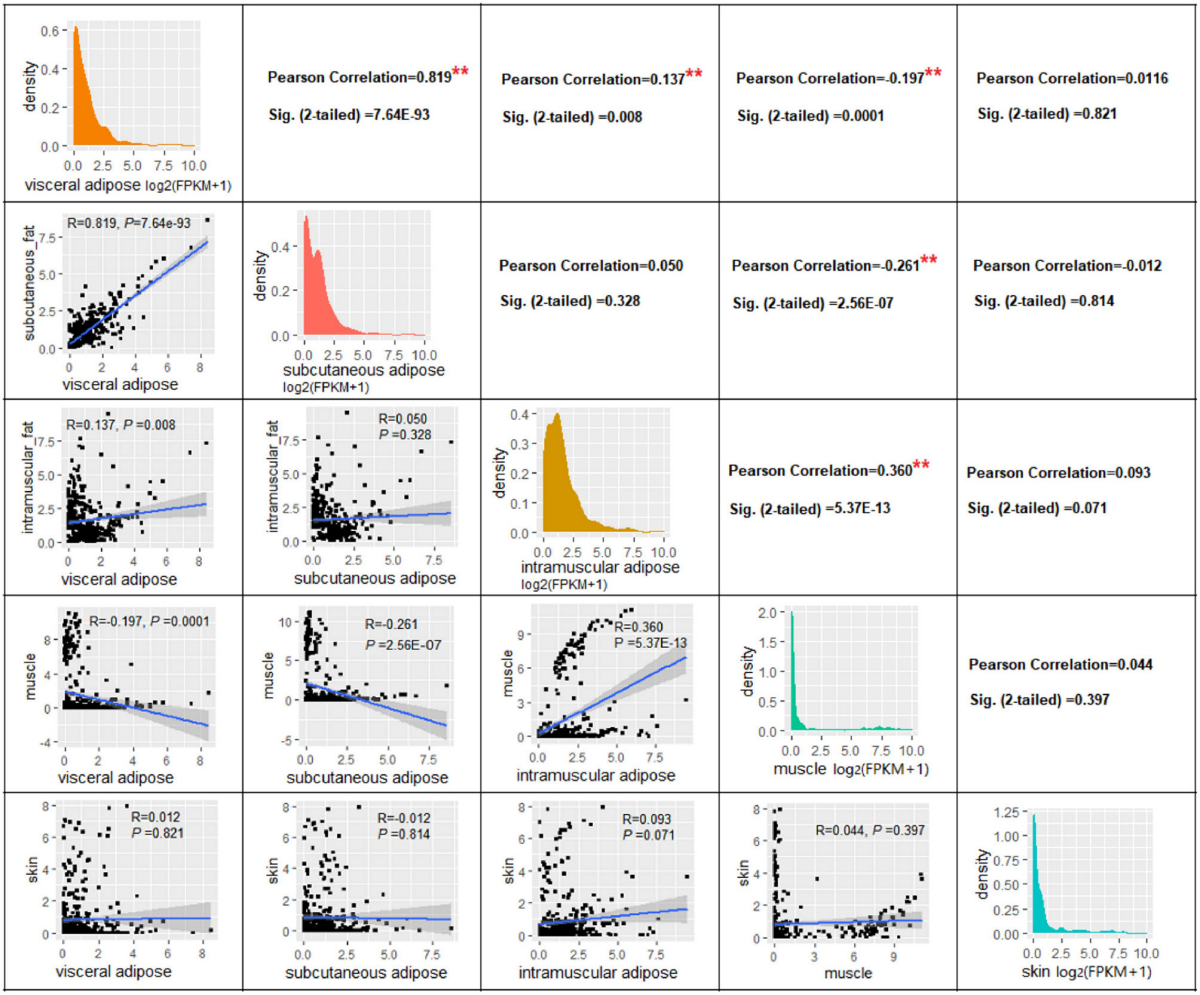
The correlation of expression level of genes in four tissues. ** *P* < 0.01.

Correlation analysis based on gene expression levels (log_2_(FPKM+1) of 378 adipose tissue-specific genes revealed a significant correlation between the VAT and SAT (*R* = 0.819, *P* = 7.64E-93), a smaller correlation coefficient between VAT and IAT (*R* = 0.137, *P* = 0.008), and surprisingly, there was no significant correlation between SAT and IAT (*R* = 0.050, *P* = 0.328) (Figure 5). Next, as expected, significant positive correlations were found between IAT and muscle tissue (*R* = 0.360, *P* = 5.37E-13), while significant negative correlations were found between muscle and SAT (*R* = −0.261, *P* = 2.556E-07), and muscle and VAT (*R* = −0.197, *P* = 0.0001) (Figure 5). This might be due to the fact that the genes we used for the correlation analysis are ATSGs, and these genes are more likely to have a positive regulatory effect on adipogenesis, but a negative effect on other tissue development.

In order to investigate whether the above correlation is due to the genes we used were ATSGs, this study used all expressed genes to conduct correlation analysis between samples. The results displayed that the correlation coefficient *R* between the three types of adipose tissues and muscle was >0.50 (*P* < 0.001, Table 2). Further analysis demonstrated that using the expression levels of all genes has a problem: some frequently expressed genes and some genes with very low expression levels have a large influence on correlation and significance analysis. Therefore, tissue-specific genes were used for correlation analysis. As a result, the correlation coefficient *R* between VAT and SAT, VAT and IAT, SAT and IAT, IAT and muscle were 0.975 (*P* < 0.001), 0.244 (*P* = 2.81E-56), 0.233 (*P* = 1.68E-51), and 0.116 (*P* = 9.68E-14), respectively. In addition, there was no significant relationship between the muscle and the other two adipose tissues. Thus, these results indicated that although the gene expression patterns in IAT and muscle were similar, IAT was still more similar to the other two adipose tissues. Furthermore, regardless of which gene group were used, the gene expression patterns of SAT and VAT were the most identical, followed by IAT.

**Table 2:** Correlation of the gene expression levels in four tissues.

**Tissues**	**Correlations**	**Visceral adipose**	**Subcutaneous adipose**	**Intramuscular adipose**	**Muscle**
Visceral adipose	Pearson correlation	1	0.975^**^	0.244^**^	0.0002
	Sig. (2-tailed)		0	2.81E-56	0.991
Subcutaneous adipose	Pearson correlation	0.979^**^	1	0.233^**^	−0.002
	Sig. (2-tailed)	0		1.68E-51	0.922
Intramuscular adipose	Pearson correlation	0.921^**^	0.931^**^	1	0.116^**^
	Sig. (2-tailed)	0	0		9.68E-14
Muscle	Pearson correlation	0.508^**^	0.525^**^	0.565^**^	1
	Sig. (2-tailed)	0	0	0	

*The data in the lower left corner are based on the analysis of all gene expression levels. The data in the upper right corner are the results of an analysis based on the expression level of tissue-specific expressed genes*.

** *P < 0.01*.

### Functional Analysis of Adipose Tissue-Specific Genes

**Figure 6:**
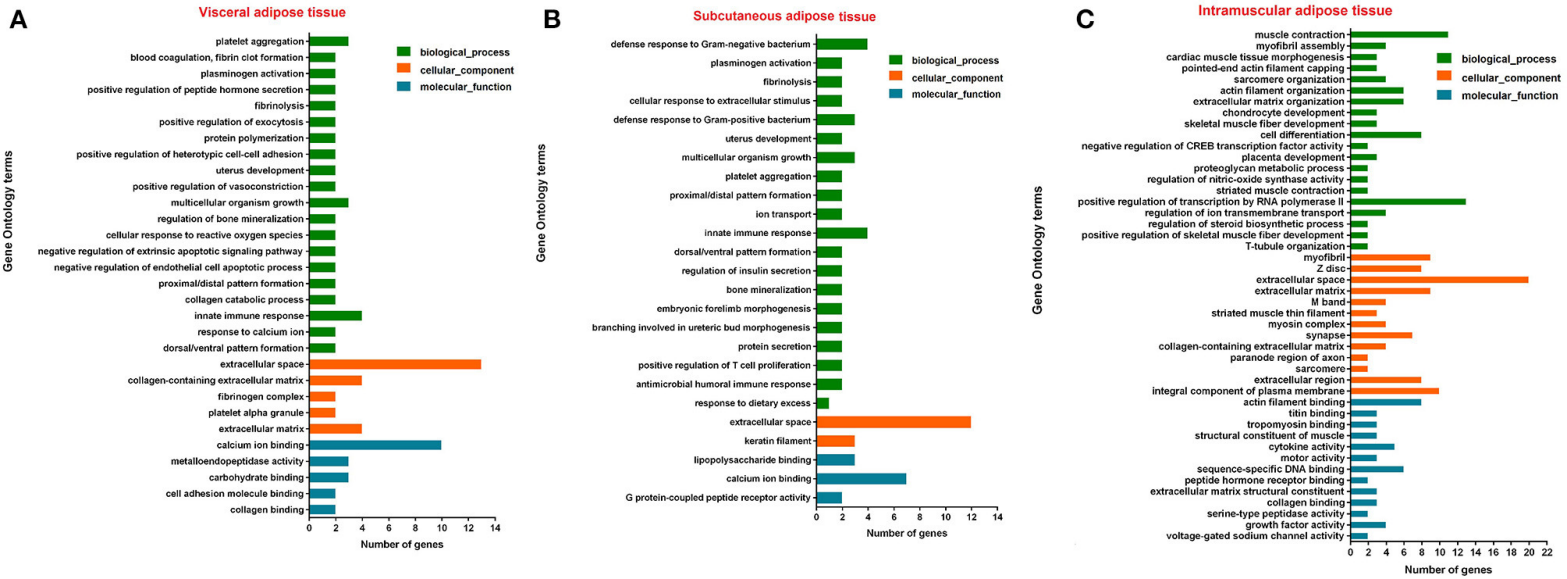
Top 20 terms of GO analysis of visceral adipose **(A)**, subcutaneous adipose **(B)**, and intramuscular adipose **(C)** tissue-specific genes (corrected *P* < 0.05).

**Figure 7:**
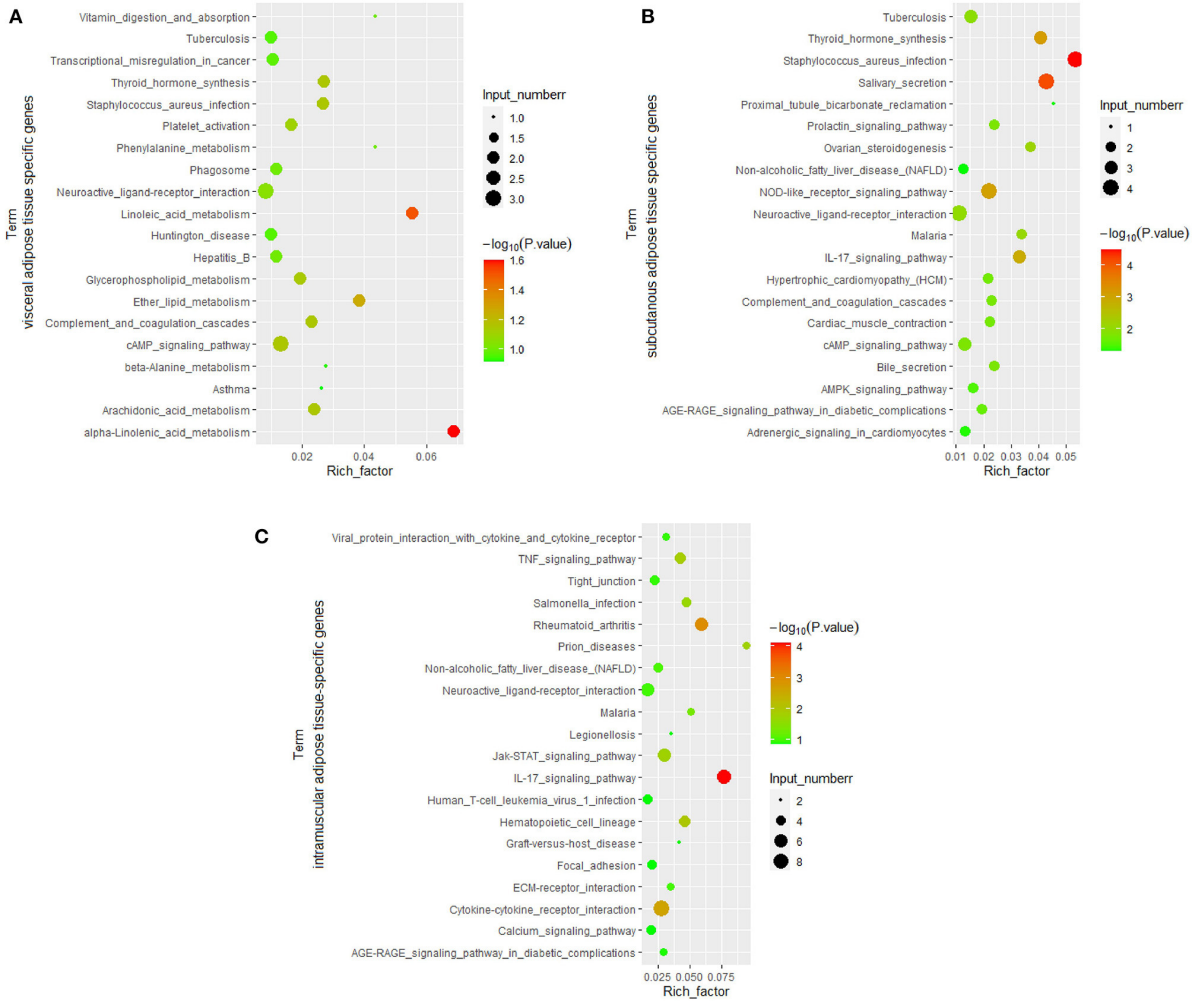
KEGG pathway analysis of adipose tissue-specific genes. **(A)**, Visceral adipose; **(B)**, subcutaneous adipose; **(C)**, intramuscular adipose.

Among tissue-specific genes, protein-coding genes account for the largest proportion, and the current annotation information on protein-coding genes is the most comprehensive. Therefore, the function of the adipose tissue-specific protein-coding genes was analyzed. Based on Go analysis, three categories were identified: molecular function, cellular component and biological process. The first 20 terms with a corrected *P* < 0.05 in biological process and all corrected *P* < 0.05 terms in the other two categories were listed (Figure 6). The KEGG analysis results were given in Figure 7, and *P*-value was the corrected *P*-value. Some terms were annotated in all three adipose tissues, such as extracellular space and calcium ion binding. Additionally, some pathways were tissue-specific, such as metalloendopeptidase activity, G protein-coupled peptide receptor activity, and actin filament binding, which may be related to tissue-specific functions.

### Genes Significantly Highly Expressed in Bovine Adipose Tissue

The above analysis identified 117, 159, and 223 genes specifically expressed in VAT, SAT, and IAT, respectively. However, the expression level of some of these genes was low. This may be because the top three genes of FPKM in all tested tissues were selected as tissue-specific genes, but some of these genes rank second or third in adipose tissue are poorly expressed. Therefore, in order to screen for genes that regulate adipose tissue function, the current study analyzed genes significantly higher expressed in adipose tissue (ATHEGs) from 52 cattle transcriptome data sets and combined them with ATSGs.

A total of 27,984 genes were detected in this study, and the differentially expressed genes in the various tissues were examined based on the number of reads of these genes. Based on the results of the correlation analysis of gene expression levels in tissues, the differential expression analysis in this part considers these three adipose tissues as an adipose tissue group and the other tissues as a non-adipose tissue group, and calculates the differentially expressed genes between the two groups. Consequently, a total of 1,575 genes were ATHEGs, while 5,840 genes had significantly lower expression in adipose tissues.

**Figure 8:**
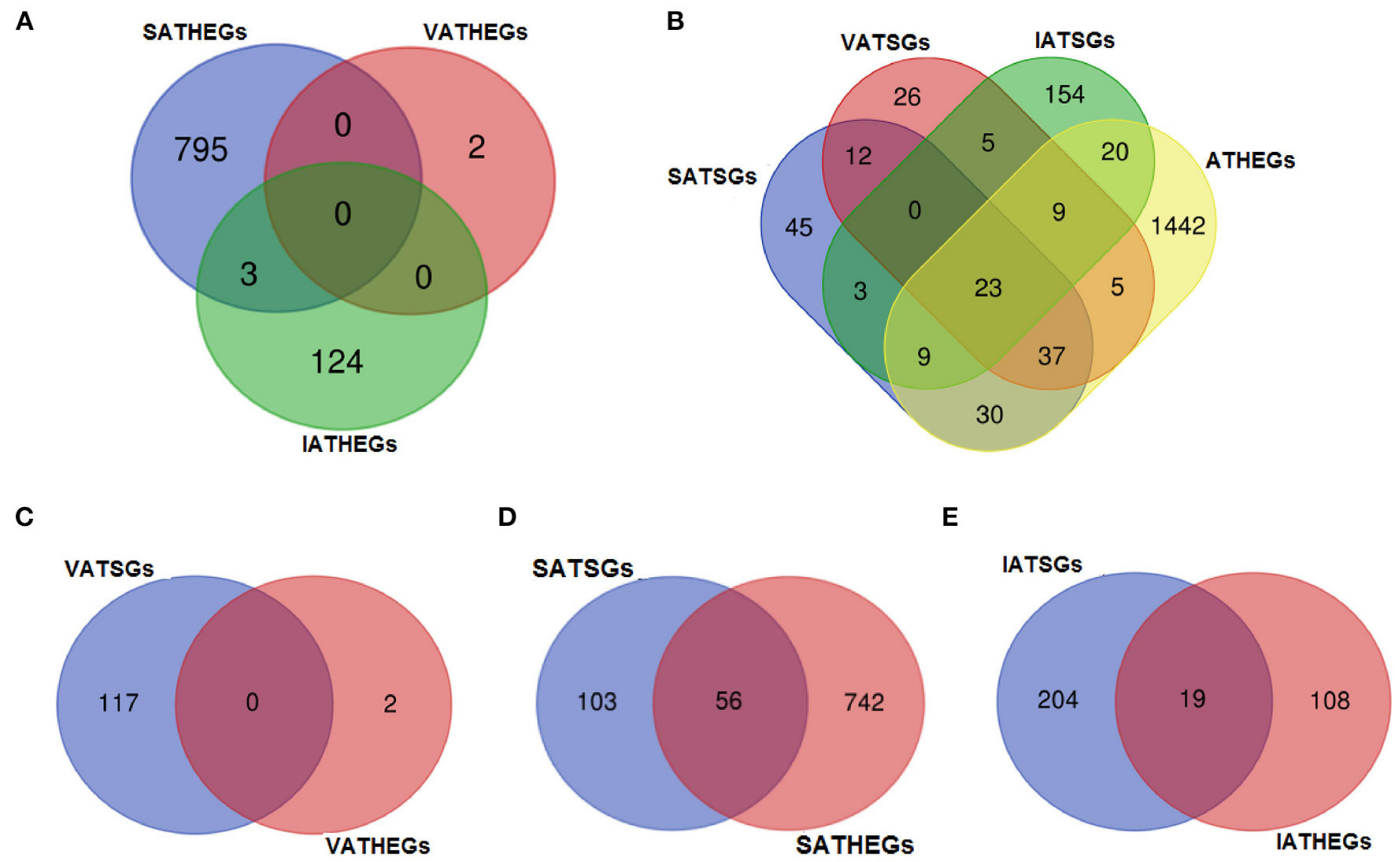
The numbers of the gene belonging to different groups. VA, visceral adipose; SA, subcutaneous adipose; IA, intramuscular adipose; HEGs, genes significantly higher expressed in tissue; TSGs, tissue-specific genes.

Furthermore, differential expression analysis of the three adipose tissues and other tissues showed that 798, 2, and 127 genes were significantly highly expressed in subcutaneous adipose (SATHEGs), visceral tissues (VATHEGs), and intramuscular adipose tissues (IATHEGs), respectively. The genes highly expressed in the three adipose were further analyzed, and no gene were found to be common among the three sets of genes. Only three genes were both SATHEGs and IATHEGs (Figure 8A). This result indicated that the gene expression patterns in the three adipose tissue types were identical, and the use of the three adipose tissue types as an adipose tissue group to analyze differentially expressed gene was reasonable.

### Statistical Analysis of Genes Highly and Specifically Expressed in Bovine Adipose Tissue

A total of 23 genes (16 protein-coding genes and seven lncRNA genes) were found to belong to both ATSGs and ATHEGs (Figure 8B). However, no genes belonging to both VATSGs and VATHEGs were detected; 56 genes belonged to SATSGs and SATHEGs; and 19 genes were a memberr of IATSGs and IATHEGs (Figures 8C–E). These tissue-specific genes with high expression in adipose tissue may be related to their specific functions and locations.

### Expression Analysis of Genes Highly and Specifically Expressed in Bovine Adipose Tissue

**Figure 9:**
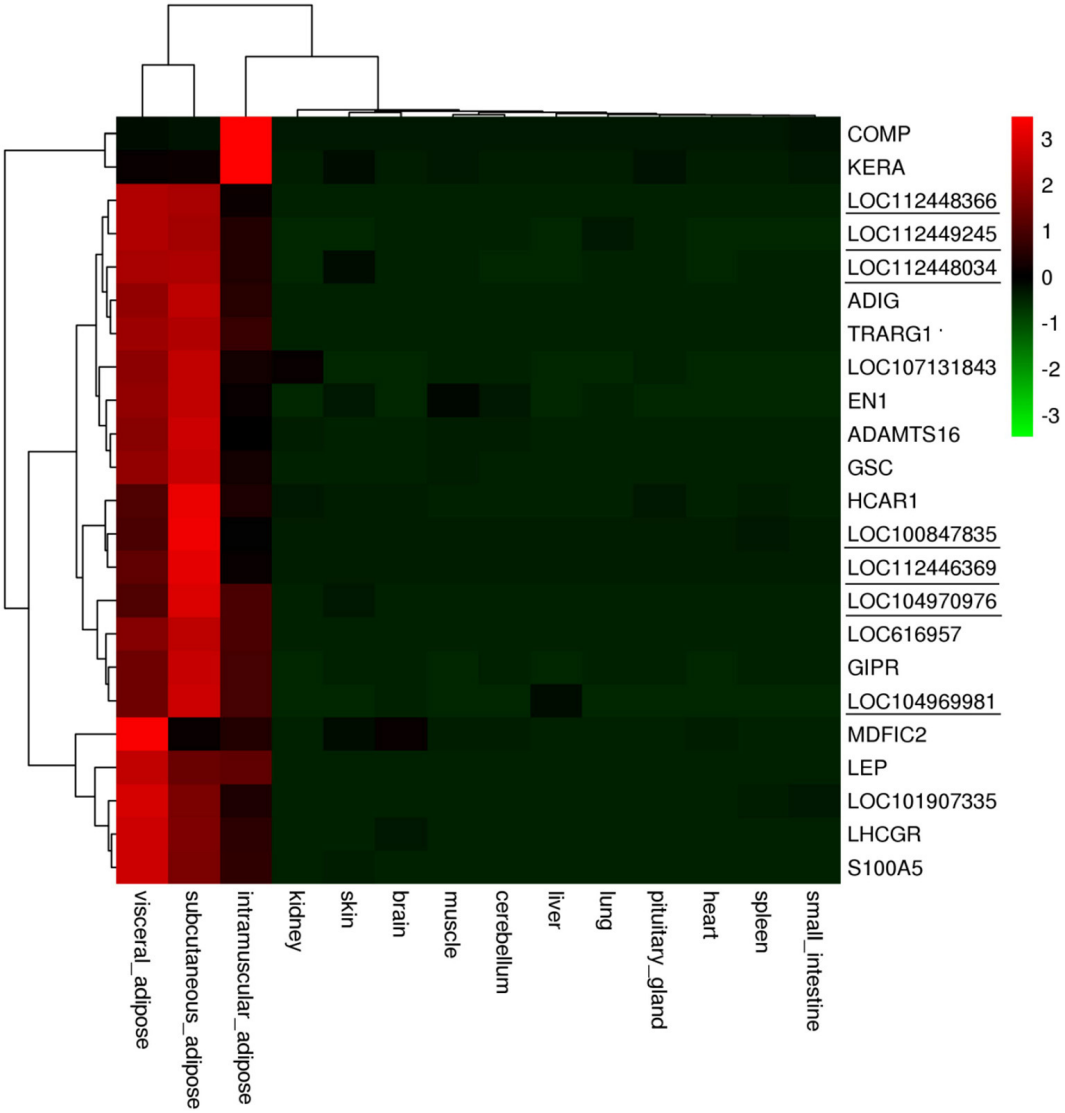
Expression levels of adipose tissue-specific and highly expressed genes in different tissues. Genes marked with underline are lncRNA genes.

**Figure 10:**
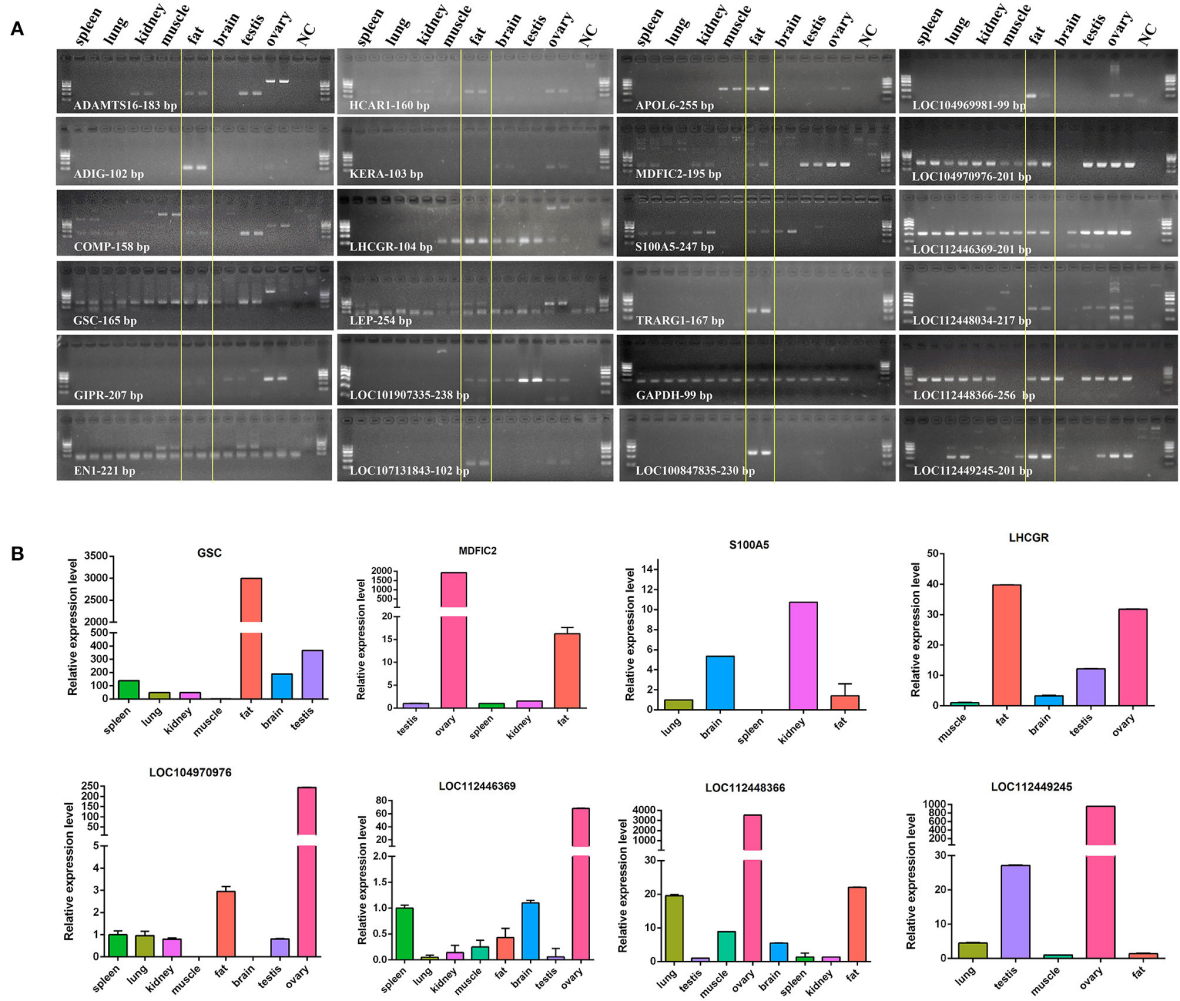
Expression profiles of genes highly and specifically expressed in tissues. **(A)**, Detected by RT-PCR; NC, H_2_O; GAPDH was internal control; The marker was Marker I: 600 - 500 - 400 (the brightest line) - 300 - 200 - 100 bp; **(B)**, detected by qRT-PCR.

In accordance with the transcriptome data of 23 genes, we made an expression profile of these genes, which displayed that these genes are highly and specifically expressed in bovine adipose tissue (Figure 9). Then, we used RT-PCR and qRT-PCR to determine the expression profiles of the 23 candidates in 8 bovine tissues, including spleen, lung, kidney, muscle, fat, brain, testis, and ovary. The results of RT-PCR showed that the mRNA expression of *TRARG1* was detected only in fat in the analyzed tissues (Figure 10A). Some other genes, such as *ADIG, HCAR1, APOL6, LOC107131843, LOC100847835, LOC104969981* were not only expressed in fat, but expressed more strongly in fat (Figure 10A). Besides, for genes expressed in more than four tissues, we detected its expression profiles using qRT-PCR. The results exhibited that GSCs had the highest expression in fat (Figure 10B). Meanwhile, several genes, involving *LHCGR, MDFIC2, LOC104970976, LOC112448366* were highly expressed in fat, but also had high expression levels in some other tissues (Figure 10B). However, a few genes, consisting of *S100A5, LOC112446369*, and *LOC112449245* are weakly expressed in fat (Figure 10B). Experimental verification results show that some candidate genes, such as *TRARG1, LOC107131843, LOC100847835*, and *LOC104969981* screened from sequencing data can be preferentially selected for the study of bovine adipose tissue development.

### Genetic Variations in the Promoter Region of the Bovine Adipose Tissue-Specific lncRNA Gene

**Figure 11:**
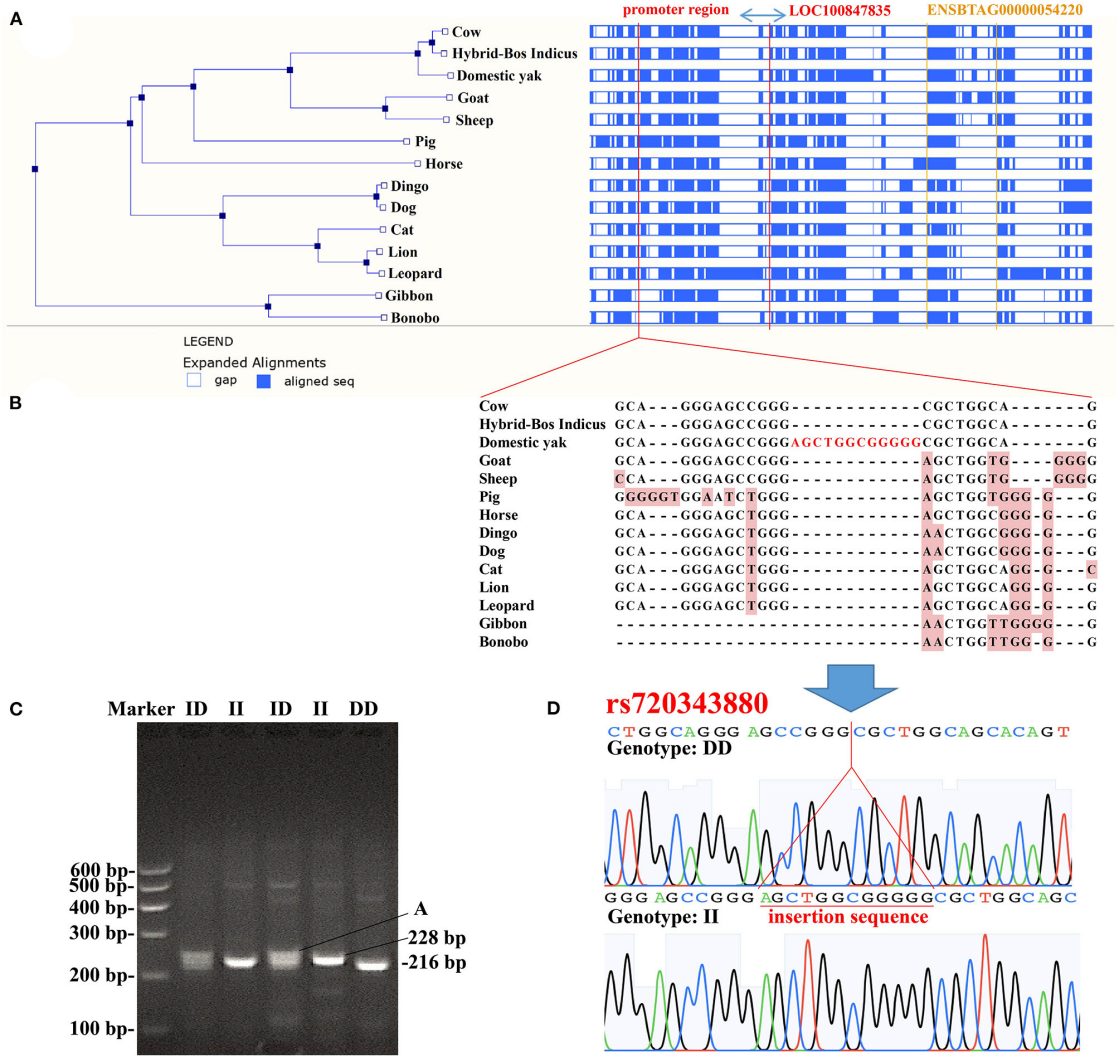
Analysis of the 12 bp indel (rs720343880) within bovine LOC100847835 promoter region **(A)**, sequences alignment of LOC100847835 and its promoter region (ENSBTAG00000054220 is a lncRNA gene noted in Ensembl database); **(B)**, Alignment of sequences containing insertion mutation (rs720343880); **(C)**, Agarose gel electrophoresis patterns of rs720343880; **(D)**, Sequencing maps of rs720343880.

The promoter is very important for gene expression. In this study, we tested a 12 bp insertion/deletion (indel) variation (rs720343880) in the promoter region of the bovine adipose tissue-specific lncRNA gene, *LOC100847835*. From the results of the sequence alignment based on the Ensembl database, it can be found that the lncRNA gene *LOC100847835* and its promoter are conserved among species, especially among ruminants (Figure 11A). Based on sequences alignment of the candidate variation (rs720343880) region, it can be seen that the 12 bp insertion sequence is fully consistent with that of the domestic yak genome (Figure 11B). The sequencing result and agarose gel electrophoresis pattern revealed that the 12 bp indel (rs720343880) does exist in the tested population of interest (Shandong black cattle, Luxi cattle, Bohai black cattle, and Mengshang cattle) (Figures 11C,D). Furthermore, genotyping results showed that there were three genotypes (deletion/deletion, DD), insertion/deletion (ID), and insertion/insertion (II) in Shandong black cattle and Luxi cattle, but only two DD and ID genotypes in Bohai black cattle and Mengshang cattle (Table 3). The results of the genetic parameters calculation demonstrated that rs720343880 was in Hardy Weinberg equilibrium in the four breeds (Table 3). The association analysis results revealed that different genotypes of rs720343880 were associated with Shandong black cattle cervical bone, bull collar, tendon, hollow bone, round small intestine, topside, hollow bone, and rump steak (Table 4). Collectively, these results mean that this variation has an important impact on cattle production.

**Table 3:** Genetic diversity of the 12 bp indel within the bovine *LOC100847835* promoter region in the four cattle breeds.

**Breeds**	**Genotypic frequencies**			**Allelic frequencies**		**Ho**	**Ne**	**PIC**	**HWE**
	**DD**	**ID**	**II**	**D**	**I**				***P*-value**
Shandong black cattle (516)	0.833	0.161	0.006	0.914	0.086	0.842	1.187	0.145	0.294
Luxi cattle (59)	0.407	0.492	0.102	0.653	0.347	0.547	1.830	0.351	0.646
Bohai black cattle (60)	0.600	0.400	/	0.800	0.200	0.680	1.471	0.269	0.500
Mengshan cattle (37)	0.811	0.189	/	0.905	0.095	0.829	1.207	0.157	0.313

*Numbers in the brackets behind the breed indicate the number of individuals*.

*Ho, observed heterozygosity; Ne, expected heterozygosity; PIC, polymorphism information content; HWE, Hardy-Weinberg equilibrium*.

**Table 4:** Association of the 12 bp indel within bovine *LOC100847835* promoter region and carcass traits of Shandong black cattle.

**Carcass traits**	**Genotypes (mean** **±SE)**		***P*-value**
	**DD**	**ID**	
**Male**
Cervical bone (kg)	11.66 ± 0.94 (*n* = 25)	14.6 ± 0.57 (*n* = 4)	0.014
Bull collar (kg)	11.86 ± 0.36 (*n* = 51)	9.93 ± 1.07 (*n* = 10)	0.042
Tendon (kg)	8.49 ± 0.88 (*n* = 27)	13.00 ± 1.00 (*n* = 4)	0.008
Round small intestine (kg)	7.69 ± 0.17 (*n* = 54)	9.00 ± 0.41 (*n* = 10)	0.004
Topside (kg)	18.91 ± 0.71 (*n* = 55)	23.33 ± 2.59 (*n* = 10)	0.029
**Female**
Hollow bone (kg)	1.06 ± 0.02 (*n* = 218)	0.98 ± 0.03 (*n* = 71)	0.020
Rump steak (kg)	5.02 ± 0.09 (*n* = 232)	4.68 ± 0.13 (*n* = 73)	0.049

## Discussion

White adipose tissue performs energy storage, protective, endocrine, and other functions. In cattle, white adipose tissue is mainly subdivided into SAT, VAT, IAT, and intermuscular adipose tissues (8). Different white adipose tissues share some common characteristics, such as the presence of lipid droplets in the cytoplasm, but they also have their own functions and characteristics. In human, excessive visceral fat deposition is a major determinant of insulin resistance, but increased subcutaneous fat improves insulin sensitivity and reduces the risk of developing type 2 diabetes (26). In cattle, excessive deposition of visceral and subcutaneous fat leads to reduced feed efficiency and poor health, but deposition of intramuscular and intermuscular fat is beneficial for improving the quality and yield of high-grade marbled beef (27). The distribution of adipose tissue varies significantly among different species, and there are also many differences between individuals of the same species. In the breeding process of beef cattle, there are advantages in depositing more adipose in the muscles, forming marbled beef, rather than excessive deposition of fat in the visceral and subcutaneous tissues. Therefore, it is of great significance to explore common and unique regulatory genes and crucial variations in cattle to breed superior beef cattle and study adipose tissue development.

The present study identified genes specifically and highly expressed in bovine adipose tissues, including protein-coding genes and non-coding genes, such as lncRNAs and miRNAs. In the process of the current construction of eukaryotic RNA sequencing libraries, rRNA removal methods are mainly achieved by enriching oligo(dT) transcription products with poly(A)-tails. In eukaryotes, partial lncRNAs and primary miRNA transcripts (pri-miRNAs) are also transcribed by RNA polymerase II and have a poly(A)-tail (3, 25). Therefore, the outcomes in this research contained both protein-coding and non-coding RNA genes.

In this survey, a total of 23 genes were found to be tissue-specific and highly expressed in adipose tissues. To the best of our knowledge, no relevant studies on the functions of the seven lncRNA have been performed. Among the 16 protein-coding genes, *LEP* gene encodes leptin (a peptide hormone), which plays an important role in regulating energy metabolism, homeostasis, endocrine, immunity, inflammatory response and reproduction. Mutations in the *LEP* gene are linked to diseases such as obesity and type II diabetes (28). Five genes are transmembrane proteins, including *ADIG, GIPR, SLC2A4, HCAR1*, and *LHCGR*. ADIG is an adipocyte specific transmembrane protein, which was critical for adipogenesis *in vivo* and *in vitro*, and ADIG knockout mice were leaner than those in control group fed a high-fat diet (29). Besides, the variations in ADIG were found to be associated with body mass index (BMI) and leptin levels (30). The GIPR gene encodes a G protein-coupled receptor, which is a duodenal hormone. The secretion of GIP is mainly induced by absorption of ingested fat (31). Wild-type mice fed a high-fat diet showed high secretion of GIP and extreme visceral and subcutaneous fat deposition and insulin resistance (32). *TRARG1* is a member of the glucose transporter family and plays an important role in glucose transport, diabetes and insulin response (33). *HCAR1* has been identified mainly in adipocytes and is involved in the anti-adipogenesis process, insulin secretion, and innate immunity (34). *LHCGR* encodes luteinizing hormone/chorionic gonadotropin receptors, a member of the G protein-coupled receptor 1 family. LHCGR is an important regulator of sexual development and reproduction in zebrafish and humans, however, its effect on adipose tissue development have not been reported (35).

*ADAMTS16* and *COMP* are extracellular matrix proteins. *ADAMTS16* can migrate to the testis, destroying collagen fibrous tissue in the fat pad of the inguinal scrotum (36). Furthermore, a mutation in *COMP* have been found to affect the balance between adipogenesis and bone formation (37). *COMP* mRNA expression level in adipose tissue and circulating COMP protein level are positively correlated with BMI/ obesity. Moreover, *COMP* expression dynamically changed during adipogenesis, and adipogenic medium supplemented with exogenous *COMP* protein promoted the differentiation of abdominal and anterior thigh adipocyte (38). EN1 and GSC are transcription factors. EN1 is expressed differently in adipose tissues of different parts of mice, with high expression in subcutaneous adipose tissues (39). In RNA-seq of 27 human tissues in NCBI database, the expression level of *GSC* in adipose tissue was significantly higher than in other tissues. The *LOC616957* (*APOL6*) gene is a member of the apolipoprotein L gene family. The miR-10b-5p was found to regulate the differentiation of 3T3-L1 adipocytes by targeting *APOL6* (40). Until now, there is no study on *LOC101907335* (antigen WC1.1-like) and *LOC107131843* (insulin receptor substrate 1-like). But study found *WC1.1* (also known as *CD163L1*), as a marker of M2-type macrophages, plays an important role in adipose tissue homeostasis (41). IRS-1 can target TAZ and inhibit adipogenic differentiation of rat bone marrow mesenchymal stem cells through PI3K-Akt and MEK-ERK pathways (42). The specific functions of the other protein-coding genes in adipogenesis are unknown.

According to Go and KEGG analysis of the candidate genes, the genes are annotated to extracellular space components and related pathways, such as focal adhesion and ECM-receptor pathways. This is closely related to the supportive function of adipose tissue and morphological changes of adipose cells during differentiation (43). In the process of adipocyte differentiation, cell morphology gradually becomes round, and this process requires not only changes in the cellular framework, but also changes in collagenase and connective tissue in the extracellular matrix to release the fixative effect on the original adipocyte morphology (44). Meanwhile, functional annotation results of candidate genes also revealed some signaling pathways with important functions in fat development, such as PI3K-Akt signaling pathway, PPAR and TNF signaling pathways.

The results of qRT-PCR showed that some genes with high expression levels both in fat and ovary, including *LHCGR, MDFIC2*, and 4 lncRNA genes (*LOC104970976, LOC112446369, LOC112448366*, and *LOC112449245*). This result may be due to the absence of ovarian tissue samples in the transcriptome data we used for tissue-specific expression gene analysis in this study. This means that the adipose tissue-specific genes identified in this study were relative to the other 11 tissues involved in this study. However, these results also reflected a close relationship between fat and the ovaries. Proper fat storage in the body is important for woman fertility. Both men and women have plenty of adipose tissue around their reproductive systems. Female mice with ovarian fat pad removal showed reduced fertility and fewer ovulated mature eggs, and the estrus cycle was significantly disrupted (45, 46). Leptin is a hormone secreted by adipose, which can affect ovarian function by interacting with gonadotropins and hormones that control gonadotropin synthesis (47). Adiponectin is also secreted by adipose. Female adiponectin deficient mice showed impaired fertility, reduced oocyte recovery, interrupted estrus cycle, increased number of atretic follicles, and impaired late follicle development (48). Multiple human studies have shown that women have higher circulating levels of adiponectin than men. This suggests that ovarian hormones may in turn regulate adiponectin production (49).

Among the seven adipose tissue-specific and highly expressed lncRNA genes, *LOC100847835* was found highly expressed in adipose tissue. From the NCBI database, we found that *LOC100847835* locates at the downstream of the *C/EBP*β gene (about 30 kb), which is a key transcription factor in adipose development (50). Since lncRNA can function through the regulation of neighboring genes, the function of *LOC100847835* may correlate with the neighboring *C/EBP*β gene. According to the data in the Ensembl database, this study revealed a 12 bp insertion variation (rs720343880) in four Chinese cattle breeds. According to the results of sequence alignment, we hypothesized that the 12 bp insertion sequence may originate from domestic yak, since gene exchange is one of the important ways for species adapt to the environment (51). Despite this, there are significant differences between yak and cattle in bone morphology, meat quality, fat content and nutritional value of meat. Thus, if the mutation derived from yak, the question is, will it affect these traits in cattle? The present study has shown that the mutation was significantly correlated with the weight of several cattle products in Shandong black cattle, but whether other aspects were influenced requires further investigation.

In conclusion, this study identified 23 adipose tissue-specific and highly expressed genes, and detected a 12 bp insertion/deletion (indel) variation (rs720343880) in the promoter region of an adipose tissue-specific lncRNA gene (LOC100847835). The different genotypes of this variation were associated with carcass traits of cattle. These results provided important outlines and hints for the study of bovine fat development, as well as the breeding of beef cattle.

## Data Availability Statement

The datasets presented in this study can be found in online repositories. The names of the repository/repositories and accession number(s) can be found in the article/Supplementary Material.

## Ethics Statement

The animal study was reviewed and approved by the Institutional Animal Care and Use Committee (IACUC) of Northwest A&F University (NWAFU-314020038), Yangling, Shaanxi, China. Written informed consent was obtained from the owners for the participation of their animals in this study.

## Author Contributions

SZ and XL conceived and designed the experiments. SZ, HX, and EJ performed the experiments. SZ and HX analyzed the data. CP, HC, FJ, ES, and XL contributed reagents, materials, and analysis tools. SZ wrote the paper. ZA revised the paper. All authors contributed to the article and approved the submitted version.

## Funding

This work was funded by the National Natural Science Foundation of China (No. 31872331) and the Agricultural Improved Seed Project of Shandong Province (2020LZGC014).

## Conflict of Interest

The authors declare that the research was conducted in the absence of any commercial or financial relationships that could be construed as a potential conflict of interest.

## Publisher's Note

All claims expressed in this article are solely those of the authors and do not necessarily represent those of their affiliated organizations, or those of the publisher, the editors and the reviewers. Any product that may be evaluated in this article, or claim that may be made by its manufacturer, is not guaranteed or endorsed by the publisher.

## Supplementary Material

Supplementary Table 1 Information for the 52 RNA sequencing data sets from bovine 14 tissues.

Supplementary Table 2 Tissue-specific genes.xls.

## Abbreviations

ATSGs: adipose tissue-specific genes
ATHEGs: genes significantly higher expressed in adipose tissue
IAT: intramuscular adipose tissue
SAT: subcutaneous adipose tissue
VAT: visceral adipose tissue
SATSGs: subcutaneous adipose tissue-specific genes
VATSGs: visceral adipose tissue-specific genes
IATSGs: intramuscular adipose tissue-specific genes
IATHEGs: intramuscular adipose tissue significant higher expressed genes
SATHEGs: subcutaneous adipose tissue significant higher expressed genes
VATHEGs: visceral adipose tissue significant higher expressed genes
FPKM: fragments per kilobase of transcript per million
PCA: principal components analysis
GO: Gene Ontology
KEGG: Kyoto Encyclopedia of Genes and Genomes
qRT-PCR: quantitative reverse transcription-polymerase chain reaction
GAPDH: glyceraldehyde-3-phosphate dehydrogenase
BMI: Body mass index
ADAMTS16: ADAM metallopeptidase with thrombospondin Type 1 Motif 16
ADIG: Adipogenin
GIPR: Gastric inhibitory polypeptide receptor or glucose-dependent insulinotropic peptide
HCAR1: Hydroxycarboxylic acid receptor 1
LHCGR: Luteinizing hormone/choriogonadotropin receptor
SMAF1: Small adipocyte factor 1
SLC2A4: Solute carrier family 2 member 4
GLUT4: Glucose transporter type 4
ECM: Extracellular matrix
EN1: Engrailed 1
APOL6: APolipoprotein L6
S100A5: S100 Calcium binding protein A5
KERA: Corneal protein
MDFIC2: MyoD Family Inhibitor Domain containing 2
IRS-1: Insulin receptor substrate 1.
