# Supplementary material for: Screening of Bovine Tissue-Specific Expressed Genes and Identification of Genetic Variation Within an Adipose Tissue-Specific lncRNA Gene
Source: Front Vet Sci. 2022 May 11;9:887520. doi: 10.3389/fvets.2022.887520 (PMC9130833; doi:10.3389/fvets.2022.887520)
Supplement: Supplementary Table 1 — Information for the 52 RNA sequencing data sets from bovine 14 tissues. [file Table_1.DOCX]

**Supplementary Table 1 |** Information for the 52 RNA sequencing data sets of bovine 14 tissues

| Tissues | SRA link ID | Tissues | SRA link ID |
| --- | --- | --- | --- |
| subcutaneous_adipose | SRR3234264 | liver | SRR10174898 |
| subcutaneous_adipose | SRR3234274 | liver | SRR10351315 |
| subcutaneous_adipose | SRR527009 | liver | SRR10351397 |
| subcutaneous_adipose | SRR527017 | liver | SRR10769820 |
| subcutaneous_adipose | SRR5363139 | liver | SRR3109705 |
| subcutaneous_adipose | SRR882031 | liver | SRR3176228 |
| subcutaneous_adipose | SRR882032 | liver | SRR3176229 |
| visceral_adipose | SRR1767622 | liver | SRR5110627 |
| visceral_adipose | SRR1767646 | liver | SRR5813941 |
| intramuscular_adipose | SRR527006 | liver | SRR8275327 |
| intramuscular_adipose | SRR527022 | liver | SRR8614592 |
| muscle | SRR10174888 | kidney | SRR10174882 |
| muscle | SRR10174890 | kidney | SRR10174883 |
| muscle | SRR4239821 | kidney | SRR3109708 |
| muscle | SRR527007 | skin_of_body | ERR3625361 |
| muscle | SRR527011 | skin_of_body | ERR3626595 |
| muscle | SRR7792116 | small_intestine | SRR10174878 |
| muscle | SRR8614367 | small_intestine | SRR8614677 |
| muscle | SRR8893823 | brain | ERR3625462 |
| muscle | SRR8893825 | brain | SRR594491 |
| heart | SRR10174870 | cerebellum | ERR3625179 |
| heart | SRR10174874 | cerebellum | ERR3626229 |
| spleen | SRR10174916 | cerebellum | SRR10948487 |
| spleen | SRR10174919 | cerebellum | SRR5363152 |
| lung | SRR10174899 | pituitary_gland | SRR1767624 |
| lung | SRR10174903 | pituitary_gland | SRR1767660 |
